# Characterization of Novel Paternal ncRNAs at the *Plagl1* Locus, Including *Hymai*, Predicted to Interact with Regulators of Active Chromatin

**DOI:** 10.1371/journal.pone.0038907

**Published:** 2012-06-19

**Authors:** Isabel Iglesias-Platas, Alex Martin-Trujillo, Davide Cirillo, Franck Court, Amy Guillaumet-Adkins, Cristina Camprubi, Deborah Bourc’his, Kenichiro Hata, Robert Feil, Gian Tartaglia, Philippe Arnaud, David Monk

**Affiliations:** 1 Servicio de Neonatología, Hospital Sant Joan de Déu, Fundació Sant Joan de Déu, Barcelona, Spain; 2 Imprinting and Cancer Group, Cancer Epigenetics and Biology Program, Bellvitge Institute for Biomedical Research, L’Hospitalet de Llobregat, Catalonia, Spain; 3 Center for Genomic Regulation, Universitat Pompeu Fabra, Barcelona, Spain; 4 Institut National de la Santé et de la Recherche Médicale, Unité de Génétique et Biologie du Développement, Institut Curie, Paris, France; 5 Department of Maternal-Fetal Biology and Department of Molecular Endocrinology, National Research Institute for Child Health and Development, Tokyo, Japan; 6 Institut de Génétique Moléculaire de Montpellier, Centre National de la Recherche Scientifique and University of Montpellier, Montpellier, France; Montana State University, United States of America

## Abstract

Genomic imprinting is a complex epigenetic mechanism of transcriptional control that utilizes DNA methylation and histone modifications to bring about parent-of-origin specific monoallelic expression in mammals. Genes subject to imprinting are often organised in clusters associated with large non-coding RNAs (ncRNAs), some of which have cis-regulatory functions. Here we have undertaken a detailed allelic expression analysis of an imprinted domain on mouse proximal chromosome 10 comprising the paternally expressed *Plagl1* gene. We identified three novel *Plagl1* transcripts, only one of which contains protein-coding exons. In addition, we characterised two unspliced ncRNAs, *Hymai,* the mouse orthologue of *HYMAI*, and *Plagl1it* (*Plagl1* intronic transcript), a transcript located in intron 5 of *Plagl1*. Imprinted expression of these novel ncRNAs requires DNMT3L-mediated maternal DNA methylation, which is also indispensable for establishing the correct chromatin profile at the *Plagl1* DMR. Significantly, the two ncRNAs are retained in the nucleus, consistent with a potential regulatory function at the imprinted domain. Analysis with catRAPID, a protein-ncRNA association prediction algorithm, suggests that *Hymai* and *Plagl1it* RNAs both have potentially high affinity for Trithorax chromatin regulators. The two ncRNAs could therefore help to protect the paternal allele from DNA methylation by attracting Trithorax proteins that mediate H3 lysine-4 methylation.

**Submitted GenBank nucleotides sequences:**

Plagl1it: JN595789

Hymai: JN595790

## Introduction

Genomic imprinting is an epigenetic form of transcriptional regulation that results in the monoallelic expression of genes from the paternal or maternal allele [1]. Currently there are around 120 confirmed imprinted genes in the mouse, with approximately 60 showing conserved imprinted expression in humans (http://igc.otago.ac.nz/home.html). Imprinted genes have been shown to play important roles in development, and code for proteins with diverse biological activities.

The allele-specific expression of imprinted genes is mediated by CpG rich sequence elements that show allelic DNA methylation [2]. These differentially methylated regions (DMRs) result from methylation deposition during oogenesis or spermatogenesis, specifically by the DNMT3A/DNMT3L *de novo* methyltransferase complex [3]–[5]. Following fertilization, the allelic methylation is maintained throughout development. In somatic tissues, most DMRs are also marked by allelic histone modifications, highlighting interplay between these two epigenetic systems [6]. Recently, non-coding RNAs (ncRNAs) have been shown to be important in recruiting histone methyltransferases to imprinted gene promoters, thus revealing the diversity of epigenetic mechanisms involved in the imprinting process [7], [8].

The *Plagl1* (also known as *Zac1*) imprinted gene maps to mouse chromosome 10. The human orthologue is located on human chromosome 6 [9], [10]. This paternally expressed gene encodes a zinc finger transcription factor with seven C_2_H_2_-type zinc-fingers that regulates apoptosis and cell cycle [11]. Loss of *PLAGL1* expression is frequently observed in many human tumours, consistent with its proposed role as a tumour-suppressor gene [12]. Over-expression of the human *PLAGL1* gene is thought to be responsible for Transient Neonatal Diabetes Mellitus (TNDM), a genetic disease characterised by severe intrauterine growth restriction and insulin dependence in neonates [13]. This over-expression can result from paternal uniparental isodisomy, paternally inherited duplications of 6q24–q25 or epigenetic mutations in which the maternal allele adopts a paternal epigenotype, resulting in biallelic expression [14]. A paternally expressed ncRNA, *HYMAI*, located in the first intron of human *PLAGL1,* is also over-expressed in TNDM patients, but the function of this transcript remains unknown [13].

To explore the mechanisms regulating *PLAGL1* imprinted expression, we performed a comparative characterisation of the orthologous domain on mouse chromosome 10. We identified numerous paternally expressed ncRNAs, which we propose may be involved in maintaining the paternal allele in a transcriptionally permissive state.

## Results

### Novel Imprinted *Plagl1* Isoforms

**Figure 1:**
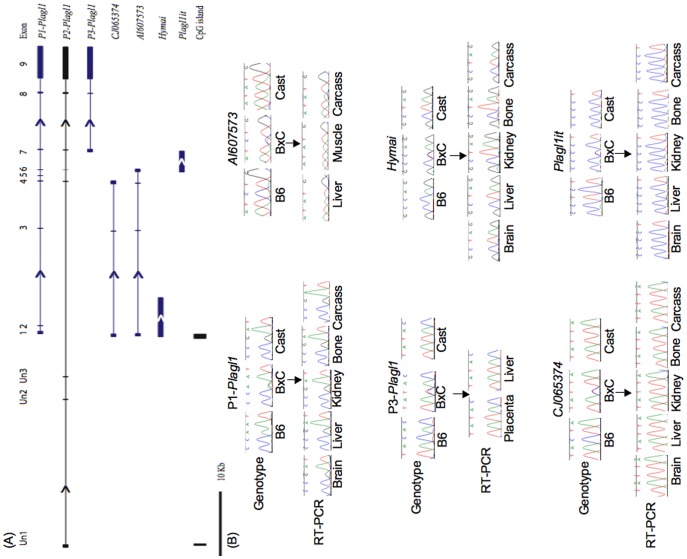
Schematic overview of the mouse chromosome 10 imprinted domain. (A) Map of the *Plagl1* locus, showing the location of the various imprinted transcripts and CpG islands (paternally expressed transcripts are in blue; biallelically expressed transcripts are in grey). Arrows represent direction of transcription. (B) The allelic expression of the various transcripts in embryonic tissues in reciprocal mouse crosses (for clarity only (B×C) F1 tissues are shown).

To first determine the size of the *Plagl1* gene in mouse, we interrogated the working draft sequence browser (NCBI26/mm8, Feb 2006). In accordance with previous reports, we find that the *Plagl1* gene covers ∼71 kb and contains 12 exons [10]. These include numerous alternatively spliced exons in the 5′UTR originating from two promoter regions embedded within two different CpG islands (Figure 1A). The majority of transcripts arise from the promoter (P1) within the DMR, whereas less abundant transcripts originate from an unmethylated CpG island ∼30 kb upstream (P2) (reference EST FJ425893). The open reading frame (ORF) for these transcripts is restricted to the last two exons, resulting in a full-length protein of 705 amino acids. All full-length transcripts share a common 3′UTR, with a polyadenylation signal 24 bp from the stop codon.

As a result of expressed sequence tag (EST) alignments, we identified three additional *Plagl1* transcripts (Figure 1A). A novel *Plagl1* transcript (reference EST BM894919) originates from a unique promoter region (P3) 5′ to the exon 7 acceptor site (gtccaag//GTCTCTT or ctcacag/GTTTGAG) of P1-*Plagl1* transcript, with a 5′UTR that extends at least 300 bp into the upstream intron mapping to an interval containing a cluster of CAGE (5′Cap Analysis Gene Expression) tags. This transcript includes the last three exons and therefore incorporates the full-length *Plagl1* ORF. The remaining two transcripts (reference ESTs CJ065374 and AI607573) originate from within the *Plagl1*-DMR region but terminate after exons 4 and 5 respectively. These different RNAs contain unique 3′UTRs, extending beyond the exon boundaries into the P1-*Plagl1* introns and do not include the *Plagl1* ORF. Northern blot analysis using a *Plagl1* exon 2–3 probe revealed, in addition to the 2 major splice variants, multiple transcripts between 700 bp and 1.7 kb (Figure S1). Using various strategically designed RT-PCR primers, we were able to confirm paternal expression of all novel *Plagl1* transcripts in RNA derived from E18.5 (B x C) F1 mouse tissues (Figure 1B).

### Conserved Expression of *Hymai* in Mouse

The human *PLAGL1* region contains the paternally expressed *HYMAI* transcript. This non-coding RNA has a transcription start site located within the *PLAGL1*-DMR. However, DNA sequence from this region shows only weak conservation between humans and mouse (data not shown) and no mouse *Hymai* is described on the UCSC sequence browser or in Genbank databases. We set out to determine whether this non-coding RNA is conserved in mouse. We utilised allelic RT-PCR amplifications restricted to intron 1 of P1-derived *Plagl1* transcript. We observed paternal expression of an RNA in various mouse tissues from E18.5 embryos (Figure 1B). Using 5′ and 3′ RACE, we were able to map the extent of this transcript, which we named ‘*Hymai’.* We identified four different transcriptional start sites (TSS) for *Hymai*, spread over a 19 bp interval embedded within the *Plagl1*-DMR (Figure S2). Using the same RACE-ready cDNA from E18.5 embryos, we were able to show that P1-*Plagl1* transcript originates from an overlapping 47 bp region, with neither P1-*Plagl1* nor *Hymai* being associated with a TATA-box. Using 3′RACE, we show that *Hymai* terminates ∼5 kb from the TSS interval, with multiple 3′ RACE products (last base chr10: 12815696 and chr10: 12815706 of mouse genome NCBI37/mm9), the longest transcript terminating 46 bp after a canonical polyadenylation signal (AATAAA). We were unable to confirm a single band on northern blot analysis, since the expression of this transcript is below the detectable limits of the technique. Analysis of the open reading frame revealed that *Hymai* has no obvious ORF (Figure S 2).

### Paternal Expression of a Novel *Plagl1* Internal Transcript, *Plagl1it*

Through examination of the UCSC sequence browser we identified 12 ESTs of various sizes transcribed from the same (+) strand as *Plagl1,* located within intron 5 of P1-*Plagl1*. The largest EST, AK087432, is 2964 bp, representing an intronless transcript with no ORF, that we named *Plagl1 intronic transcript* (*Plagl1it*) (Figure 1B; Figure S2). Using RACE, we found that this transcript initiates within intron 5 of P1*-Plagl1* and is at least 3.6 kb, with its 5′ end overlapping the 3′UTR of the paternally expressed EST AI607573 by ∼400 bp. Northern blot analysis confirmed the presence of a faint band of between 3.5–kb (Figure S1). Using RACE and RT-PCR we were unable to link *Plagl1it* to *Plagl1*, confirming this is an independent overlapping transcript and not an alternative *Plagl1* exon or UTR (Figure S2). Using allele-specific RT-PCR, we were able to show that this transcript is expressed solely from the paternal chromosome in different mouse tissues (Figure 1B).

### Expression of *Hymai* and *Plagl1it* is Uniformly Low Throughout Development

Next, we set out to analyse the tissue-specificity of expression for the novel transcripts. Using quantitative RT-PCR we determined the abundance of the transcripts in placenta, brain and decapitated embryos at E11.5, E12.5, E14.5, E18.5 and in addition to brain, liver, kidney and muscle from both newborn and adult mice (Figure S1). We observed that *Plagl1* expression was consistently higher than both *Hymai* and *Plagl1it* in all tissues and developmental stages analysed. All genes show a marked decrease in expression after birth, in both newborn and adult tissues.

### The ncRNAs are Nuclear Retained, Unstable Transcripts

**Figure 2:**
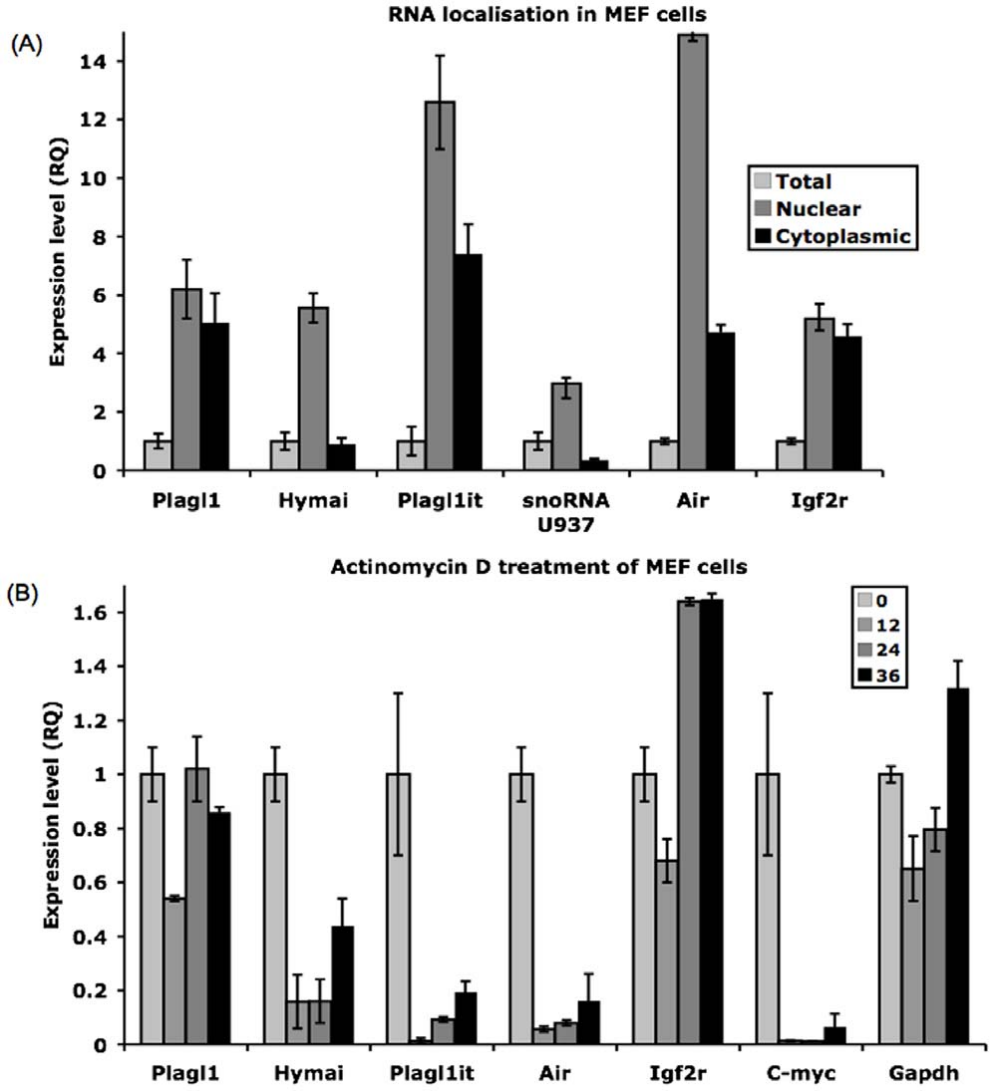
Cellular localization and RNA stability of the ncRNAs. (A) Distribution of the various transcripts in the nuclear (dark grey) and cytoplasmic (black) fractions, compared to total RNA (light grey). *U937 snoRNA* and *Airn* are nuclear-retained controls, whereas *Igf2* is cytoplasm-exported control. (B) Abundance of the various transcripts after exposure to Actinomycin D to determine RNA stability. The relative expression values of the control untreated samples are set to 1 (light grey bars) for each transcript. *C-Myc* and *Airn* are control transcripts for with short half-life; *Gapdh* and *Igf2r* are long half-life controls.

As a first step to explore whether *Hymai* and *Plagl1it* could have functional roles, we determined the cellular localisation of these ncRNAs. We performed qRT-PCR on nuclear, cytoplasmic and total RNA isolated from mouse embryonic fibroblasts (MEF) cells. The efficiency of the nuclear separation was confirmed using the *U937 snoRNA* and paternally expressed *Airn* ncRNAs that have been shown previously to not be exported to the cytoplasm. We observed residual *Airn* in the cytoplasmic fraction, suggesting slight nuclear RNA contamination only detectable when analysing highly expressed nuclear retained transcripts. The *Igf2r* mRNA was used as a control for a transcript that is exported to the cytoplasm [15]. Quantitative RT-PCR analysis revealed that the *Plagl1* transcript is efficiently exported to the cytoplasm for translation, whereas the *Hymai* ncRNA is retained in the nucleus. The *Plagl1it* transcript is present in both the nucleus and cytoplasm, but is more abundant in the nuclear fraction (Figure 2A).

To determine the stability of *Hymai* and *Plagl1it* in MEFs, actinomycin (ActD) was used to inhibit transcription. We used *C-Myc* and the unspliced *Airn* transcripts as controls for RNAs with short half-life and *Gapdh* and *Igf2r* as control for RNAs with long half-lives [8], [15]. Figure 2B shows that after 12 hours treatment with ActD the *C-Myc* and *Airn* mRNAs are largely depleted, whereas *Gapdh* and *Igf2r* are not affected. The *Plagl1* transcript remains abundant under these ActD conditions, suggesting that it is a highly stable transcript. However, both *Hymai* and *Plagl1it* are diminished after 12 hours to levels that are similar to *C-Myc* and *Airn,* indicating that these ncRNAs are unstable transcripts.

### DNMT3L is Indispensable for *Hymai*, *Plagl1it* and *Plagl1* Imprinting

**Figure 3:**
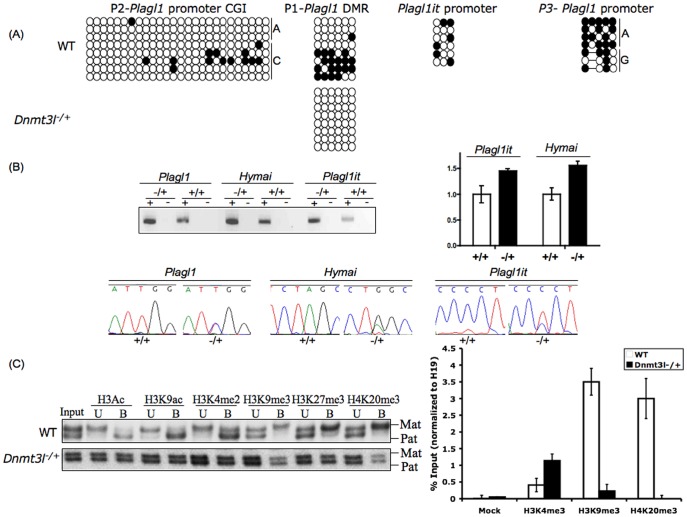
Analysis of *Plagl1* region in *Dnmt3l −/+.* (A) The methylation status of the *Plagl1* promoter regions in wild type +/+ and *Dnmt3l* −/+ embryos examined by bisulphite PCR. Each circle represents a single CpG dinucleotide on a DNA strand, a methylated cytosine (•) or an unmethylated cytosine (○). (B) RT-PCRs on cDNA generated with (+) and without (−) reverse transcriptase show an increase in the expression of the imprinted transcripts in *Dnmt3l*−/+ embryos as a result of reactivation of the maternal allele. (C) The histone modification signature of the *Plagl1*-DMR in wild type B×C embryos, and after targeted deletion of the *Dnmt3l* gene. DNA extracted from antibody bound (B) and unbound (U) chromatin fractions were subject to either qPCR or PCR and SSCP analysis with primers that can discriminate parental alleles.

DNA methylation inherited from the maternal germline requires the DNMT3L/DNMT3A complex [3], [4]. Using bisulphite DNA sequencing, we were able to confirm that the CpG island overlapping the P1-*Plagl1* and *Hymai* transcription start sites is differentially methylated, whereas P2-*Plagl1* arises from an unmethylated CpG island. The promoters of *Plagl1it* and P3-*Plagl1* initiate from regions of low CpG content that display partial, but not allelic DNA methylation (Figure 3A). To assess if the maternal allelic silencing of *Hymai*, *Plagl1it* and the various *Plagl1* transcripts requires maternal germline DNA-methylation, we used qRT-PCR on mouse embryos that had inherited a deletion of the *Dnmt3l* gene from a homozygous mutant mother [3]. Lack of this essential imprinting factor led to the loss of maternal methylation at the *Plagl1*-DMR, and increased expression of all transcripts in targeted E8.5 embryos due to reactivation of the maternal allele (Figure 3B).

### The *Plagl1*-DMR Chromatin Profile Requires Allelic DNA Methylation

Recent studies have suggested that there is a mechanistic link between DNA and histone methylation at imprinted DMRs [6]. To determine if there was a link between allelic DNA-methylation and any histone modifications present at the *Plagl1*-DMR, we first looked for the presence of modifications by allelic chromatin immunoprecipitation on whole embryos followed by discrimination of the parental alleles in the precipitated chromatin fractions. Our analysis focused on different modifications of histone H3 and H4; pan-acetylation of H3, acetylation of H3 lysine-9 (H3K9ac) and H3 lysine 4 dimethylation (H3K4me2) as markers of active chromatin; and the repressive marks of H3 lysine 9 trimethylation (H3K9me3) and H3 lysine 27 trimethylation (H3K27me3), along with the histone H4 lysine 20 trimethylation (H4K20me3).

We ascertained allelic enrichment using a polymorphic region between inbred mouse strains that maps within 200 bp of the CpG island associated with the *Plagl1*-DMR. Within this region H3K4me2 and H3K9ac were strongly enriched specifically on the unmethylated paternal allele (Figure 3C). The same regions showed precipitation of the repressive markers H3K9me3, H3K27me3 and H4K20me3 on the DNA-methylated maternal allele. We extended our analysis to include the promoter regions of P2-*Plagl1*, which maps within an unmethylated CpG island, and *Plagl1it,* whose promoter is not associated with a CpG island. In both cases, we failed to detect allelic precipitation, suggesting that the presence of allelic histone modifications is restricted to the DMR region (data not shown).

To assess whether the allelic histone modifications we observe at the *Plagl1*-DMR require the maternally derived DNA methylation, we performed allelic ChIP on *Dnmt3l* −/+ embryos. In agreement with observations at other imprinted DMRs [6], we detect a dramatic effect on histone modification distribution, with the lack of allelic enrichment due to “paternalization” of the maternal allele, as a result of increased H3K4me3 and a concomitant reduction of H3K9me3 and H4K20me3 (Figure 3C).

### *Hymai* and *Plagl1it* Potentially Interact with Active Chromatin Regulatory Factors

To determine whether *Hymai* and/or *Plagl1it* could be involved in maintaining the active state of the paternal allele of the *Plagl1*-DMR, we performed a prediction of their interaction propensities against four Trithorax proteins (ASH1/KMT2H, MLL1/KTM2A, WDR5, CFP1) using the recently published catRAPID method [16]. CatRAPID allows evaluation of the interaction potential of polypeptides and RNAs using their physiochemical properties, with initial studies revealing high interactions propensities for the ncRNAs *Xist* and *HOTAIR* with Polycomb repressive complex proteins (interaction propensities 76–99% and 69–99%, respectively). In addition, CatRAPID was able to accurately predicted RNA binding of the human RNase P proteins (interaction propensities 68–99%) and discriminate RNA binding (interaction propensity >65%) and non-binding (interaction propensity <5%) proteins of the human ribonuclease mitochondrial RNA processing (MRP) complex [16].

**Figure 4:**
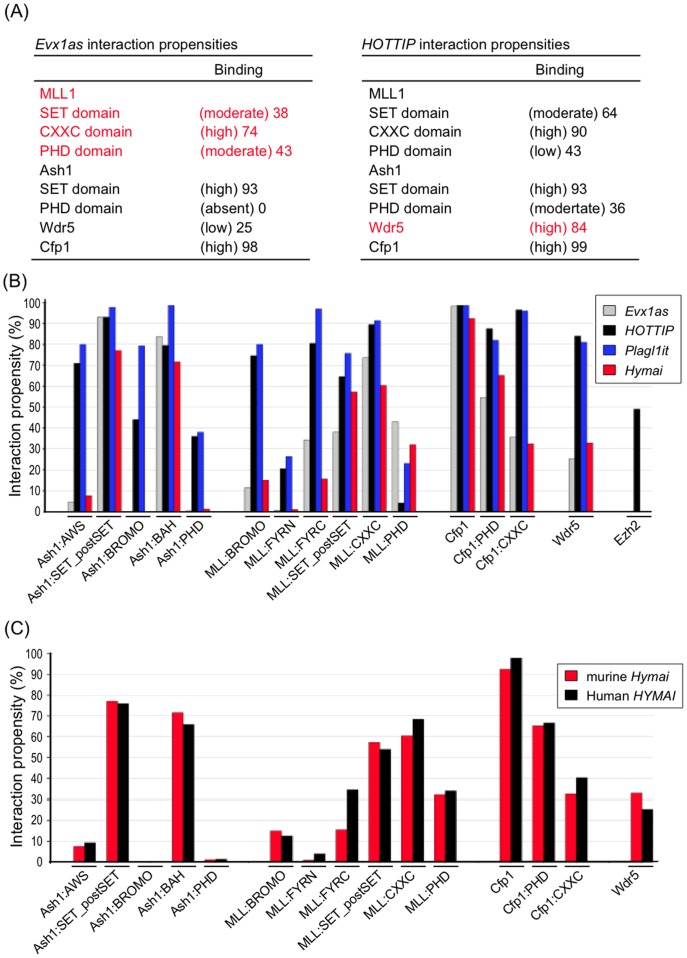
CatRAPID analysis of ncRNA-protein interactions. (A) CatRAPID analysis reveals the interaction propensities of the control ncRNAs; *Evx1as* with MLL1/KTM2A and *HOTTIP* with WDR5. (B) The interaction propensities for the control ncRNAs and for *Hymai* and *Plagl1it* with various components (and sub-domains) of the H3K4 and H3K27 methylation machinery. (C) Similar ncRNA-protein interactions revealed by CatRAPID analysis for *Hymai* (in black) and the human orthologue *HYMAI* (in red).

In our analysis we used ncRNAs *Evx1as* and *HOTTIP* as controls because they are known from experimental work to directly recruit MLL1 and WDR5 proteins to *HOX* gene loci [17], [18]. We observed moderate to high interaction propensities between *Evx1as* and various functional domains of the MLL1 protein, and between *HOTTIP* and WDR5 (Figure 4A). Interestingly both are predicted to interact strongly with the CFP1 PHD and Ash1 SET-postSET regions. Subsequent analysis using our imprinted ncRNAs revealed that *Hymai* and *Plagl1it* are highly prone to interaction; in particular they have strong binding propensity with Trithorax proteins. We observe that *Hymai* and *Plagl1it* have negligible propensity for interaction with the Polycomb repressive complex protein EZH2, which trimethylates H3K27 to repress transcription (Figure 4B). Finally, we compared the interaction propensities for *Hymai* and the human orthologue *HYMAI*. We observe that despite having different sequences, and *HYMAI* being subject to splicing, the two transcripts have similar potential interactions (Figure 4C), with 3′ regions having the highest interaction propensities (data not shown). Overall the murine *Hymai* could interact with MLL1 slightly less than human *HYMAI*, but both display high interaction propensities for ASH1 SET-postSET domains and for CFP1 (Figure 4C). Taken together, our results suggest that both *Hymai* and *Plagl1it* may interact with chromatin machinery that confers a permissive chromatin state.

## Discussion

Here we show a detailed investigation of the genomic organisation of the mouse *Plagl1* domain. As in humans, *Plagl1* transcripts can originate from multiple promoters, one of which is a DMR previously shown to be methylated in the female germline and therefore likely to be the ICR for this region [10], [19]. A second alternative promoter located ∼30 kb upstream is within an unmethylated CpG island. This promoter is orthologous with the human P2-*PLAGL1* which gives rise to biallelically expressed transcripts in lymphocytes and pancreas [20]. In mouse, transcription from this promoter is low in somatic tissues, however the primary function of this promoter may be to allow transcription across the P1-*Plagl1* promoter CpG island in growing oocytes. This has been proposed to be important for the establishment of the allelic DNA-methylation at this DMR [21]. In addition to the alternative transcripts of *Plagl1*, we show the presence of two additional ncRNAs, *Hymai* and *Plagl1it*. In keeping with other reported ncRNAs, these are expressed at a lower level than nearby mRNAs, consistent with the hypothesis that ncRNAs may fulfil a regulatory function [22]. We were able to successfully map the TSS and polyadenylation sites for both *Hymai* and *Plagl1it* using RACE-ready cDNAs, indicating that these transcripts comprise rare ncRNAs that are polyadenylated and have 5′-Caps. The reason for the nuclear enrichment of these ncRNA is unknown, as the majority of polyadenylated RNAs are exported to the cytoplasm [23], [24]. However, the lack of RNA splicing may be a significant factor in the nuclear retention, as has been described for the various full-length and spliced isoforms of *Airn* [16] and other mRNAs [24].

The precise roles of *Hymai/HYMAI* and *Plagl1it* are unclear, but it is likely that they have a different function to the other known imprinted long ncRNAs such as *Airn* and *Kcnq1ot1* due to their different affinities for chromatin remodelling enzymes. *Airn* and *Kcnq1ot1* have been shown to attract histone methyltransferases G9a/KTM1C and EZH2/KMT6, and are involved in *cis-*silencing of nearby genes [8], [24], [25]. However, recent studies demonstrated that large ncRNAs can also guide the permissive H3K4 histone methyltransferase machinery to target genes in mouse ES cells and MEFs [17], [18] and can act as local enhancers [26]. Thus, unlike other imprinted “repressive” ncRNAs, our data suggests that *Hymai* and *Plagl1it* could act to keep the paternal allele unmethylated and in a transcriptionally permissive state. In fitting with this hypothesis, we observe that *Hymai* and *Plagl1it* are unstable transcripts, which presumably ensures they stay near the site of transcription, preventing their action in *trans* on the maternal allele within the same nucleus. Our *in silico* analysis using catRAPID suggests that *Plagl1it* and the mouse and human *Hymai/HYMAI* may interact with various components of the Trithorax group proteins, with potentially the highest specificity for SET-proSET and zinc finger CXXC domains, in agreement with previous *in vitro* experiments showing that these domains can bind RNA [27], [28]. *In vitro* demonstration of these interactions is technically challenging since *Hymai* and *Plagl1it* are not expressed at the levels required for RNA-ChIP in MEF cells. However, we observe that WDR5 does precipitate preferentially on the paternal unmethylated allele of the *Plagl1*-DMR (Figure S3) substantiating our hypothesis.

### Conclusions

Germline loss of methylation at the maternal allele of the *PLAGL1*-DMR is known to result in TNDM [13], [29]. In addition, *PLAGL1* has been suggested to play a role in numerous cancers, including ovarian, breast and pituitary adenomas, with somatic deletions or gains in methylation resulting in loss of expression of this tumour suppressor gene [30]. We hypothesise that the newly identified ncRNA could potentially guide the H3K4 methylation machinery to the paternal allele of the *PLAGL1*-DMR, and thus protect this region from pathological hypermethylation.

## Materials and Methods

### Mouse Crosses and Cell Lines

For the analysis of expression, wild type mouse embryos and placentas were produced by crossing C57BL/6 (B) with *Mus musculus castaneus* (C) mice. RNA and DNA from *DNMT3L^−/+^* mice (B×C) was isolated and extracted as previously described [3]. Animal husbandry and breeding were licensed by Direction Departementale des Services Veterinaires (authorization number 34–104). Homozygous C57BL/6 mice of various gestational ages were used for expression analysis. Mouse embryonic fibroblast cell lines were established from both wild-type (B x C) F1 (Bourc’his laboratory) and C57BL/6 (B) with *Mus musculus molossinus* (JF1) F1 (Feil laboratory) mice. The Institutional Review Board of Bellvitge Institute for Biomedical Research granted scientific and ethical approval for this study (PR232/09).

### RNA Preparations

Total RNA from (B×C) F1 wild type embryos, *Dnmt3l*−/+ embryos and MEF cells was isolated using Trizol reagent (Invitrogen) and subjected to double DNase 1 treatment to ensure preparations were free of contaminating DNA. 1 ug of RNA was used for first strand cDNA synthesis using Promega reagents according to the manufacturer’s instructions. Nuclear and cytoplasmic RNA was isolated from MEF cells using the Norgen kit (Biotek corporation, Ontario, Canada) following manufacturers instructions. cDNA was generated using 0.5 ug of cytoplasmic, nuclear and total RNA.

### Actinomycin Treatment

5×10^5^ MEF cells seeded per 10 cm dish were cultured for 36 hrs. At time point 0, the medium was removed; cells were washed with PBS and then incubated with medium supplemented with 10 mg/ml Actinomycin D (dissolved in ethanol). At each time point (0, 12, 24 and 36 hrs) cells from a treated dish were harvested for RNA using Trizol (Invitrogen).

### 5′ and 3′ RACE

Mouse E18.5 embryo Marathon-Ready cDNA (Clontech) was used for RACE using the Advantage 2 polymerase kit (Clontech). The PCR step was performed with the gene-specific primers located in ESTs for *Plagl1* and *Plagl1it* in combination with nested adaptor oligonucleotides following manufacturers recommendations. The PCR products were subcloned into pGEM T-easy vector (Promega) and 20 colonies were sequenced using an ABI prism 3100 DNA sequencer (Applied Biosystems). The full-length sequences of Plagl1it and Hymai have been deposited in GenBank and have been assigned the accession numbers JN595789 and JN595790 respectively.

### Northern Blot Analysis

To determine the size of *Plagl1it, Hymai* and the truncated *Plagl1* transcripts, we used custom-made northern blots containing 20 µg of total RNA extracted from CD1 embryos (Zyagen, San Diego, USA). The blots were hybridised with an *β-Actin* probe prior to use to confirm equal loading. Unique sequences for each transcript were amplified by PCR, and the resulting amplicon probes were radiolabelled with (^32^P)CTP using the Ready-To-Go DNA labelling Beads (Amersham). Hybridizations were carried out overnight at 65°C and washed according to manufacturer’s instructions.

### RT-PCR Conditions

Allelic RT-PCRs, reactions were performed using primers that flanked polymorphisms. The amplification cycle numbers for each transcript were determined to be within the exponential phase of the PCR, which varied for each gene, but was between 32–42 cycles. The subsequent amplicons were sequenced using both the forward and reverse primers (Table S1 for primer sequences).

### Real-time RT-PCR

All PCR amplifications were run in triplicate on a 7900 Fast real-time PCR machine (Applied Biosystems) following the manufacturers’ protocol. All primers were optimized using SYBR Green (see additional data file 5 for primer sequences) and melt curve analysis to ensure that amplicons were specific and free of primer-dimer products. Thermal cycle parameters included Taq polymerase activation at 95°C for 10 min for 1 cycle, repetitive denaturation at 95°C for 15 sec, and annealing at 60°C for 1 min for 40 cycles. All resulting triplicate cycle threshold (Ct) values had to be with 1 Ct of each other. The quantitative values for each triplicate were determined as a ratio with the level of *Gapdh* expression (B-actin for actinomycin experiments), which was measured in the same sample, and then averaged to provide relative expression values.

### Analysis of Allelic DNA-methylation

Approximately 1 µg DNA was subjected to sodium bisulphite treatment and purified using the EZ GOLD methylation kit (ZYMO, Orange, CA). Bisulphite PCR primers for each region were used with Hotstar Taq polymerase (Qiagen, West Sussex, UK) at 40 cycles and the resulting PCR product cloned into pGEM-T easy vector (Promega) for subsequent sequencing (see Table S1 for primer sequences).

### Chromatin Immunoprecipitation (ChIP)

ChIP was carried out on wild type embryos, MEF cells and *Dnmt3l* −/+ embryos. ChIP was performed as previously described [6] using the following Upstate Biotechnology antisera directed against H3ac (06-599), H3K9ac (07-352), H3K4me2 (07-030), H3K9me3 (060904589), H3K27me3 (07-449) and H4K20me3 (07-463) (Upstate Biotechnology). DNA extracted from precipitated chromatin fractions was PCR amplified, and parental alleles were discriminated by either SSCP (*PLAGL1*-DMR) or by direct sequencing. Polymorphisms within 1 kb of the CpG islands were identified by interrogating SNP databases or through genomic sequencing (see Table S1 for primer sequences and location). Only ChIP sample sets that showed enrichment for additional imprinting control regions were used in the analysis. Precipitation levels in the ChIP samples were determined by real-time PCR amplification, using SYBR Green PCR kit (Applied Biosystems). Each PCR was run in triplicate and results are presented as percentage precipitation and normalised to the level of the *H19*-DMD, since methylation at this paternally methylated DMR is unaffected after maternal transmission of the Dnmt3l deleted allele.

### catRAPID Analysis

We employed the catRAPID algorithm to predict potential interactions between ncRNAs and proteins [16]. This algorithm was trained using RNA-protein pairs described in the NPInter database. We calculated the average interaction propensity of each RNA species (fragmented into ∼1 kb segments because of sequence length restrictions) against complete protein and unique functional domains. Multiple domains adjacent in sequence were joined together (e.g. three PHD domains in MLL1 and SET/proSET regions). In the case of domain association with a size <50 amino acids additional flanking amino acids were added upstream and downstream.

## Supporting Information

Figure S1 (**A**) Expression of *Plagl1*, *Hymai* and *Plagl1it* in various tissues from embryos at different gestational stages (e = embryonic day; NB = new born). (**B**) Northern blot analysis using probes specific for *Plagl1* exon 2–3, *Plagl1it* and *Hymai*. A single transcript of less than 4 kb is detected for *Plagl1it* consistent with RACE and RT-PCRs results. Truncated *Plagl1* transcripts, between 700–1.7 kb, correspond to CJ065374 and AI607573.
(TIF)

Figure S2 **Mapping of the RACE products to determine the extents of the novel transcripts and open reading frame analysis.** (A) The overlapping start sites for P1-*Plagl1* and *Hymai*. (B) analysis for open reading frame using DNA Strider for *Hymai*. (C and D) The 5′ and 3′ ends of *Plagl1it* in relation to *Plagl1* transcripts, and ORF analysis.
(TIF)

Figure S3 **Chromatin immunoprecipitation of WDR5 in MEF cells.** (A) The upper panel shows PCR amplification of the β-actin promoter control region and *Plagl1*-DMR in the WDR5-ChIP. The lower panel is the genotypes of the input and IP (B x C), showing preferential precipitation of the paternal allele compared to input as calculated from relative area under the nucleotide curve at the SNP position. (B) Confirmation of preferential paternal enrichment by *Hinf*1 RFLP analysis.
(TIF)

Table S1 **Table of PCR primer sequences.**
(DOC)
