# Supplementary figures and images for: Characterization of Novel Paternal ncRNAs at the Plagl1 Locus, Including Hymai, Predicted to Interact with Regulators of Active Chromatin
Source: PLoS One. 2012 Jun 19;7(6):e38907. doi: 10.1371/journal.pone.0038907 (PMC3378578; doi:10.1371/journal.pone.0038907)

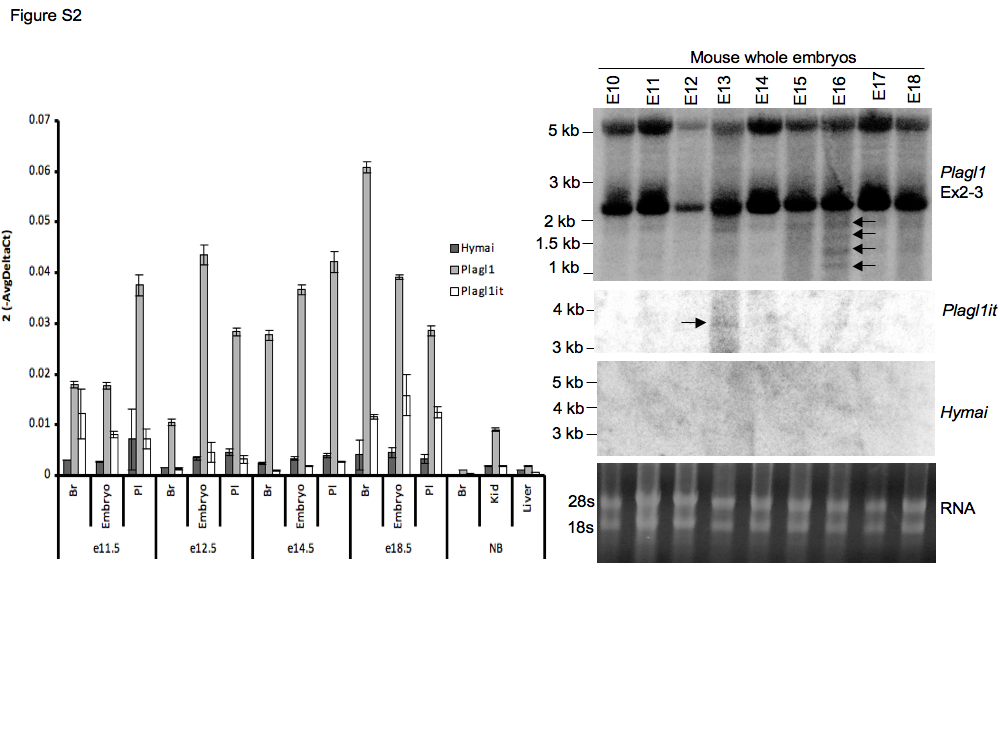

Supplement: Figure S1 — (A) Expression of Plagl1, Hymai and Plagl1it in various tissues from embryos at different gestational stages (e = embryonic day; NB = new born). (B) Northern blot analysis using probes specific for Plagl1 exon 2–3, Plagl1it and Hymai. A single transcript of less than 4 kb is detected for Plagl1it consistent with RACE and RT-PCRs results. Truncated Plagl1 transcripts, between 700–1.7 kb, correspond to CJ065374 and AI607573. (TIF) [file pone.0038907.s001.tif]

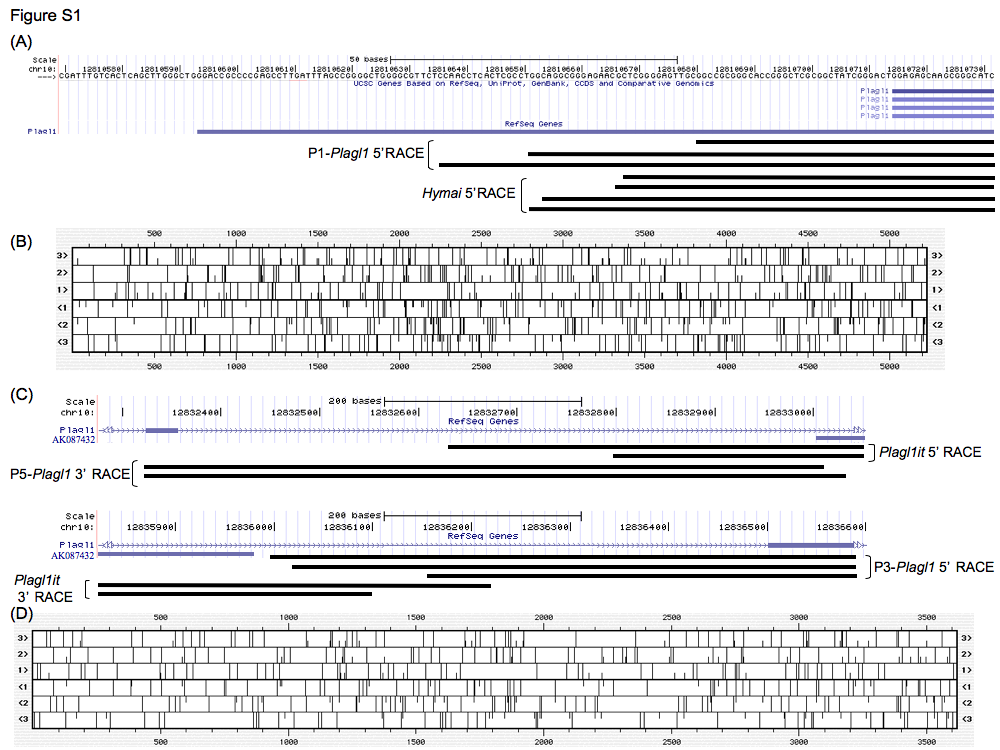

Supplement: Figure S2 — Mapping of the RACE products to determine the extents of the novel transcripts and open reading frame analysis. (A) The overlapping start sites for P1-Plagl1 and Hymai. (B) analysis for open reading frame using DNA Strider for Hymai. (C and D) The 5′ and 3′ ends of Plagl1it in relation to Plagl1 transcripts, and ORF analysis. (TIF) [file pone.0038907.s002.tif]

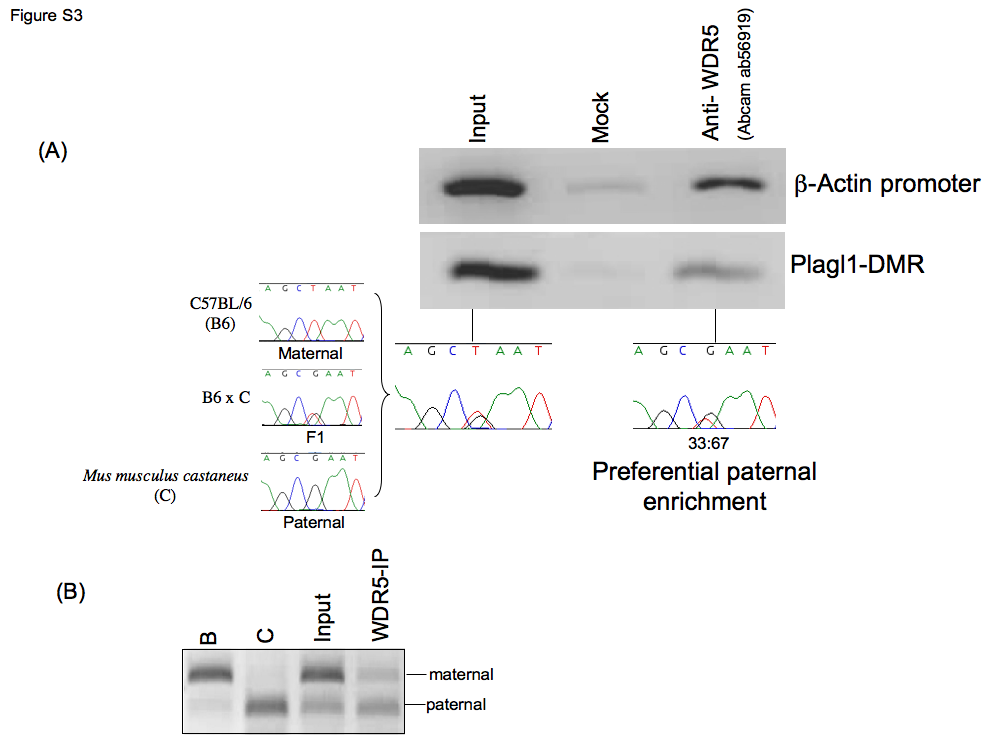

Supplement: Figure S3 — Chromatin immunoprecipitation of WDR5 in MEF cells. (A) The upper panel shows PCR amplification of the β-actin promoter control region and Plagl1-DMR in the WDR5-ChIP. The lower panel is the genotypes of the input and IP (B x C), showing preferential precipitation of the paternal allele compared to input as calculated from relative area under the nucleotide curve at the SNP position. (B) Confirmation of preferential paternal enrichment by Hinf1 RFLP analysis. (TIF) [file pone.0038907.s003.tif]
