# Supplementary material for: Characterization of Novel Paternal ncRNAs at the Plagl1 Locus, Including Hymai, Predicted to Interact with Regulators of Active Chromatin
Source: PLoS One. 2012 Jun 19;7(6):e38907. doi: 10.1371/journal.pone.0038907 (PMC3378578; doi:10.1371/journal.pone.0038907)
Supplement: Table S1 — Table of PCR primer sequences. (DOC) [file pone.0038907.s004.doc]

| **Primer Name** | **Sequence** | **Polymorphism Identification Number** |
| --- | --- | --- |
| *Plagl1it* promoter ChIP F | taagggcttaaaataaccctgtg | rs33585018 |
| *Plagl1it* promoter ChIP R | gactgaaaccaatctgtcaaaaac |  |
| *Plagl1it* qRT-PCR F | gcaaccccacacatccttaagc |  |
| *Plagl1it* qRT-PCR R | gaacattcacagaaactcaagg |  |
| *Plagl1it* RT-PCR F | gcaaccccacacatccttaagc | rs33580060; rs33580063; rs33581097 |
| *Plagl1it* RT-PCR R | gaaaatgaccctgtgaaattaag |  |
| Hymai qRT-PCR F | aagtagtgacaaccggggccat |  |
| Hymai qRT-PCR R | gaacacaaatcacctcttccc |  |
| Hymai RT-PCR F | cactcgcctggcaggcgggag | rs50316897; rs29364824 |
| Hymai RT-PCR R | gaacacaaatcacctcttccc |  |
| *Plagl1* 3’UTR RT-PCR F | ggccacacactcgccacagaag | rs29315063; rs33583470 |
| *Plagl1* 3’UTR RT-PCR R | ccaccatcaagcccgttctcg |  |
| *Plagl1* qRT-PCR F | gattgcttcagcgtgccatcg |  |
| *Plagl1* qRT-PCR R | actcctctgactcctatgcaaa |  |
| *Plagl1* P3 RT-PCR F | gcatgtaggacaaaggatatac |  |
| *Plagl1* P3 RT-PCR R | ccaccatcaagcccgttctcg | rs33583470 |
| *Plagl1* exon 1 RT F | cactcgcctggcaggcgggag |  |
| *Plagl1* P4 RT-PCR R | gtgttcagtcttcagatctcacactg | rs33579258; rs33579260 |
| *Plagl1* P5 RT-PCR R | cctggggactgtactctagctc | rs3358018 |
| *Plagl1it* promoter bisulphite Out F | gagtttatagggttggaagtg |  |
| *Plagl1it* promoter bisulphite In F | gttggagatttagtggaggttgg |  |
| *Plagl1it* promoter bisulphite In R | aaacccaccaaaaaaattc |  |
| *Plagl1it* promoter bisulphite Out R | ctctttattaacatttaaacccacc |  |
| *Plagl1* P2 CGI bisulphite F | gtaggttttagagggtttggg |  |
| *Plagl1* P2 CGI bisulphite R | ttataaaatacacacaaaaac |  |
| *Plagl1* P1 DMR bisulphite F | tttgggttaggataggagaaagagtgt |  |
| *Plagl1* P1 DMR bisulphite In R | cttaaaaacaatttttataacc |  |
| *Plagl1* P1 DMR bisulphite Out R | cccctataaaaaccttattc |  |
| *Plagl1* P2 CGI ChIP F | cagagcacatggatccgctca | rs48357717; rs33576132 |
| *Plagl1* P2 CGI ChIP R | cagagcagccttgctcgcccagg |  |
| *Plagl1* P1 ChIP DMR F | cgcacatttagctactaataca | rs29330747 |
| *Plagl1* P1 ChIP DMR R | caatttttgtggccaaaggct |  |
| C-myc qRT-PCR F | agctgtttgaaggctggatttcctt |  |
| C-myc qRT-PCR R | ggtgtctcctcatgcagcactagggg |  |
| Gapdh qRT-PCR F | cactgaagggcatcttgggctacac |  |
| Gapdh qRT-PCR R | tcattgtcataccaggaaatgagctt |  |
| Air qRT-PCR F | ggctcagcaaacagcacca |  |
| Air qRT-PCR R | gcctgtgattgctcagttattcc |  |
| Igf2r qRT-PCR F | tgtgcagttacacatgggaagct |  |
| Igf2r qRT-PCR R | agtgagtcagggactgagcg |  |
| U73A snoRNA F | aacatcagaaaaatgtaatgttatcagtggc |  |
| U73A snoRNA R | tcatgtgatgagaaactgtttcggtcc |  |
| B-actin qRT-PCR F | cctgacggccaggtcatcac |  |
| B-actin qRT-PCR R | ggagcaatgatcttgatcttc |  |
| Utrn RT-PCR F | gtttgcaaaatgaccttgaag |  |
| Utrn RT-PCR R | cccattttgctcccggagggttg |  |
| Stx11 RT-PCR F | gaggtgtcttcacacacagc |  |
| Stx11 RT-PCR R | gctcgaacatgtcctcaatc |  |
| Sf3b5 RT-PCR F | cgtctttctcccgcgcctgcac |  |
| Sf3b5 RT-PCR R | ctcgaaatgcgcatcactctc |  |
| Ltv1 RT-PCR F | gatgacccagaaaacctccttg |  |
| Ltv1 RT-PCR R | catcaagctcatttgttggc |  |
| Phactr2 RT-PCR F | ggccaggttgacacctgcagac |  |
| Phactr2 RT-PCR R | ctgtttcccttgtagagcgc |  |

Primers used for allelic expression, qRT-PCR, bisulphite PCR and ChIP.
